# GLP-1 receptor agonists are a transformative prehabilitation tool for weight loss in obese patients undergoing elective hernia repair

**DOI:** 10.1007/s00464-024-11308-6

**Published:** 2024-10-05

**Authors:** Graham J. Spurzem, Ryan C. Broderick, Patricia Ruiz-Cota, Hannah M. Hollandsworth, Bryan J. Sandler, Santiago Horgan, Eduardo Grunvald, Garth R. Jacobsen

**Affiliations:** 1 Department of Surgery, Division of Minimally Invasive Surgery, University of California San Diego, San Diego, CA USA; 2 Division of General Internal Medicine, Bariatric and Metabolic Institute, University of California San Diego, San Diego, CA USA

**Keywords:** Glucagon-like peptide 1, GLP-1, Obesity, Hernia, Prehabilitation, Weight loss

## Abstract

**Background:**

Obesity is an independent risk factor for complications after abdominal hernia repair. Glucagon-like-peptide-1 (GLP-1) receptor agonists are gaining popularity as pharmacologic weight loss adjuncts and may help patients reach weight loss goals for surgery. We examine our early experience utilizing GLP-1 agonists versus lifestyle modifications alone to achieve weight loss in patients before elective hernia repair.

**Methods:**

This single-center, retrospective review identified obese patients who underwent elective hernia repair from 2014 to 2023. Patients were asked to achieve a BMI ≤ 33 kg/m^2^ before surgery. Patients who lost weight with GLP-1 therapy in addition to lifestyle changes were compared to a control cohort that achieved similar preoperative weight loss without GLP-1 therapy. Primary outcome was mean time from GLP-1 agonist initiation and initial surgery clinic visit to surgery. Secondary outcomes were 30-day morbidity, mortality, and reoperation rates, and hernia recurrence.

**Results:**

Forty-six patients with ventral/incisional, flank, umbilical, parastomal, inguinal, and hiatal hernias were identified (GLP-1 *N* = 24, control *N* = 22). 81.8% (*N* = 18) of controls had a ventral/incisional hernia, compared to 45.8% (*N* = 11) of GLP-1 patients (*p* = 0.03). Mean BMI at GLP-1 agonist initiation was similar to mean BMI at initial clinic visit for controls (38.1 ± 4.9 vs 38.2 ± 2.7 kg/m^2^, *p* = 0.66). Preoperative mean percentage total weight loss (14.9 ± 7.5 vs 12.4 ± 6.9 kg, *p* = 0.39) and mean BMI reduction (6.0 ± 3.8 vs 4.9 ± 2.3 kg/m^2^, *p* = 0.43) were similar between groups. The mean time from GLP-1 agonist initiation to surgery was significantly shorter than initial clinic visit to surgery for controls (6.3 ± 4.0 vs 14.7 ± 17.6 months, *p* = 0.03). There was no statistically significant difference in time from initial clinic visit to surgery between groups (7.6 ± 4.4 vs 14.7 ± 17.6 months, *p* = 0.06). There was no significant difference in 30-day morbidity between groups (8.3 vs 27.3%, *p* = 0.13).

**Conclusion:**

GLP-1 agonists accelerate preoperative weight loss for obese hernia patients without negatively impacting postoperative outcomes.

Abdominal wall hernias are common, with an estimated prevalence of 1.7% for all ages. Inguinal hernias account for approximately 75% of abdominal wall hernias, with a lifetime risk of 27% in men and 3% in women [1]. Ventral hernias (VH) are also highly prevalent. It is estimated that primary VH occur in approximately 20% of adults and incisional VH develop in up to 30% of midline abdominal incisions [2, 3]. An estimated 611,000 ventral and 1 million inguinal hernia repairs are performed each year in the United States (US), adding several billion dollars in annual health care expenditure [4]. Abdominal wall hernias also have the potential to cause significant morbidity, and hernia repair poses a substantial challenge in surgical practice.

Obesity, defined as a body mass index (BMI) greater than 30 kg/m^2^, is an independent risk factor for hernia development, recurrence, and complications after hernia repair [5–7]. Obesity increases intra-abdominal pressure, and the pro-inflammatory state of adipose tissue is thought to have adverse effects on wound healing, increasing the risk of subsequent incisional hernia formation [8]. One study of 313 patients who underwent abdominal wall reconstruction for VH repair with a mean follow-up of 15.6 months found that the risk of hernia recurrence was significantly increased in patients with higher BMI [9]. Patients with a BMI of 30–34.9 kg/m^2^ had a recurrence rate of 29.8%, compared to 8.3 and 12.5% for patients with BMIs of 15–24.9 kg/m^2^ and 25–29.9 kg/m^2^ respectively. Patients with obesity desiring hernia repair are often asked to meet weight loss goals before surgery in order to mitigate these risks. Lifestyle modifications, including changes in diet, physical activity, and behavioral therapy, are the cornerstone of weight loss [10]. However, pharmacologic therapy has emerged as an important adjunct to obesity management. Several weight loss medications have been approved by the US Food and Drug Administration (FDA), and other off-label agents are currently in use [11].

Although initially approved for the treatment of type 2 diabetes mellitus, glucagon-like peptide 1 (GLP-1) receptor agonists have moved into the mainstream as pharmacologic adjuncts for weight loss, with proven safety and efficacy in patients with and without diabetes [12]. The first drug approved for weight management by the FDA was liraglutide 3.0 mg with once daily administration [13]. Semaglutide 2.4 mg was approved by the FDA in June 2021. Semaglutide has achieved greater efficacy compared to liraglutide through slight structural changes, and its pharmacokinetic properties enable once weekly dosing [14]. Other drugs of the same class, such as dulaglutide and tirzepatide, also have demonstrated weight loss benefits [15, 16]. The mechanism of weight loss with GLP-1 agonists is thought, at least in part, to be due to slowed gastric emptying and increased satiety through effects on appetite centers in the brain [17].

There is a growing body of literature examining the utility of GLP-1 agonists as a tool for perioperative weight management in surgical patients, particularly in bariatrics. It has been shown that GLP-1 agonists can be an effective therapy for weight regain after bariatric surgery [18]. A recent study demonstrated that GLP-1 agonist use in bariatric patients results in significantly more weight loss prior to surgery, without increased time to surgery or complication rates [19]. Little has been studied about the effectiveness of GLP-1 agonist therapy in obese patients who desire hernia repair. The goal of this study is to examine the safety and efficacy of using GLP-1 agonists for weight loss in the prehabilitation of obese patients undergoing elective hernia repair. We hypothesize that GLP-1 agonists will accelerate preoperative weight loss compared to lifestyle modifications alone, decreasing the time to surgery without increasing complications.

## Methods

### Study design

A retrospective, single-center review of a prospectively maintained IRB-approved database was performed identifying patients with obesity who underwent elective hernia repair from 2014 to 2023. Patients were prescribed GLP-1 agonists specifically for weight loss in addition to lifestyle changes before elective hernia repair beginning in 2021. Patients with a BMI > 33 kg/m^2^ and a ventral, incisional, flank, umbilical, parastomal, inguinal, or hiatal hernia were included in the analysis. Patients previously prescribed GLP-1 agonists for diabetes management were excluded. For comparison to patients who received GLP-1 therapy, patients with a BMI > 33 kg/m^2^ and one of the aforementioned hernia types who achieved a similar amount of weight loss before surgery without GLP-1 agonists were identified. Both patient cohorts were managed by a multidisciplinary weight loss team comprised of surgeons, obesity medicine physicians, and registered dieticians in our comprehensive Bariatric and Metabolic Institute. Our program offers individual consultations to develop personalized weight management strategies, including diet/exercise plans and weight loss medications. Patients in the program were generally asked to achieve a BMI of 33 kg/m^2^ or less prior to hernia repair.

Patients prescribed GLP-1 agonists for weight loss were actively managed by a dedicated obesity medicine physician. The decision to initiate and continue these medications was based on shared decision-making discussions, the discretion of the obesity medicine physician, and insurance coverage. Insurance coverage was an important factor when deciding whether to initiate GLP-1 treatment and when choosing a particular drug. All patients who were started on a GLP-1 agonist and underwent surgery continued treatment for the duration of their preoperative course.

Demographic data included age, gender, Charlson Comorbidity Index (CCI), preoperative weight and BMI, GLP-1 medication used, other concurrently used weight loss medications, time from either GLP-1 agonist initiation or initial surgery clinic visit to surgery, and hernia type. Operative data included hernia defect size, wound classification, operative approach, and need for component separation (either Rives-Stoppa with retrorectus dissection (RR), anterior component separation with external oblique release (EOR), or posterior component separation with both RR dissection and transversus abdominis muscle release (TAR)).

### Outcomes

The primary outcome was mean time from GLP-1 agonist initiation or initial surgery clinic visit to surgery. For each cohort, mean percentage total weight loss (%TWL) and mean BMI reduction were calculated over these time periods. Baseline weight for the weight loss calculations was either weight at GLP-1 agonist initiation or weight at initial surgery clinic visit. Secondary outcomes were 30-day morbidity, mortality, and reoperation rates and hernia recurrence. Interval follow-up time and hernia recurrence were determined by either the last noted physical exam involving the abdomen or last recorded abdominal imaging in the electronic medical record. Superficial surgical site infection was defined as an infection involving the skin and subcutaneous tissue of the incision. Postoperative ileus was defined as obstipation and intolerance of oral intake > 3 days after surgery.

### Statistical analysis

All statistical analyses were performed in R (Version 4.1.2, Vienna, Austria). For categorical variables, Fisher’s exact test was used for analyses with small samples, otherwise Pearson’s chi-square test was used. For continuous variables, Wilcoxon rank-sum test was used for non-parametric data, otherwise independent sample *t*-test was used. Time-to-hernia surgery was also assessed for each cohort with a log-rank test described by Kaplan–Meier curves. A *p*-value of < 0.05 was considered statistically significant.

## Results

### Patient demographics and operative data

A total of 24 patients with obesity who were prescribed GLP-1 agonists for weight loss before elective hernia repair were identified. 22 patients with obesity who achieved similar weight loss before hernia surgery without GLP-1 agonists (control group) were identified for comparison. Of the 24 patients in the GLP-1 group, 21 (87.5%) received semaglutide, 2 (8.3%) received dulaglutide, and 1 (4.2%) received liraglutide. One patient (4.5%) in the control group was taking phentermine during follow-up. 81.8% of patients in the control group had a ventral/incisional hernia (*N* = 18), compared to 45.8% (*N* = 11) in the GLP-1 group (*p* = 0.03). The GLP-1 group also included 7 (29.2%) umbilical hernias, 2 (8.3%) hiatal hernias, 1 (4.2%) flank hernia, and 1 (4.2%) parastomal hernia, while the control group was solely comprised of ventral/incisional, umbilical, and inguinal hernias. There were otherwise no significant differences between the groups with regard to age, gender, CCI, hernia defect size, wound class, operative approach, or need for component separation (Table 1).

**Table 1:** Patient demographics and operative data

	GLP-1 (*N* = 24)	Control (*N* = 22)	*p*-value
Age (years), mean (SD)	56.5 (11.6)	61.8 (11.1)	0.12
Female, *n* (%)	16 (66.7)	13 (59.1)	0.82
CCI, mean (SD)	1.8 (1.4)	2.5 (1.5)	0.12
Hernia type, *n* (%)			
Ventral/incisional	11 (45.8)	18 (81.8)	**0.03**
Umbilical	7 (29.2)	2 (9.1)	0.14
Inguinal	2 (8.3)	2 (9.1)	0.99
Hiatal	2 (8.3)	0	0.49
Flank	1 (4.2)	0	0.99
Parastomal	1 (4.2)	0	0.99
Hernia defect size (cm^2^), mean (SD)	57.7 (72.8)	88.3 (115.5)	0.62
Wound classification, *n* (%)			
Class 1	23 (95.8)	20 (90.9)	0.60
Class 2	0	2 (9.1)	0.22
Class 3	1 (4.2)	0	0.99
Class 4	0	0	–
Operative approach, *n* (%)			
Open	12 (50.0)	11 (50.0)	0.99
Laparoscopic	3 (12.5)	7 (31.8)	0.16
Robotic	9 (37.5)	4 (18.2)	0.20
Component separation, *n* (%)	9 (37.5)	9 (40.9)	0.99

Bold values indicate statistical significance

*GLP-1* glucagon-like peptide-1,* CCI* Charlson Comorbidity Index,* SD* standard deviation

### Preoperative weight loss and time to surgery

Mean weight at GLP-1 agonist initiation (GLP-1: 106.5 ± 15.3 kg vs control: 102.1 ± 15.6 kg, *p* = 0.34) and mean BMI at GLP-1 agonist initiation (GLP-1: 38.1 ± 4.9 kg/m^2^ vs control: 38.2 ± 2.7 kg/m^2^, *p* = 0.66) for the GLP-1 group were not significantly different from mean weight and BMI at initial surgery clinic visit for the control group. Preoperative mean %TWL (GLP-1: 14.9 ± 7.5 vs control: 12.4 ± 6.9, *p* = 0.39) and mean BMI reduction (GLP-1: 6.0 ± 3.8 kg/m^2^ vs control: 4.9 ± 2.3 kg/m^2^, *p* = 0.43) were also not significantly different between groups. There were no significant differences between mean weight/BMI or preoperative %TWL/BMI reduction between the GLP-1 and control groups at initial clinic visit (Table 2).

**Table 2:** Preoperative weight loss and time to surgery

Metric, mean (SD)	GLP-1 (*N* = 24)	Control (*N* = 22)	*p*-value
Weight (kg)			
GLP-1 agonist initiation	106.5 (15.3)	–	0.34
Initial surgery clinic visit	106.8 (13.7)	102.1 (15.6)	0.28
BMI (kg/m^2^)			
GLP-1 agonist initiation	38.1 (4.9)	–	0.66
Initial surgery clinic visit	38.1 (4.5)	38.2 (2.7)	0.99
Weight (kg) at time of surgery	90.7 (16.2)	89.0 (14.2)	0.71
BMI (kg/m^2^) at time of surgery	32.1 (3.9)	33.1 (2.5)	0.31
Preoperative %TWL			
GLP-1 agonist initiation	14.9 (7.5)	–	0.39
Initial surgery clinic visit	15.3 (8.9)	12.4 (6.9)	0.33
Preoperative BMI reduction (kg/m^2^)			
GLP-1 agonist initiation	6.0 (3.8)	–	0.43
Initial surgery clinic visit	6.1 (4.1)	4.9 (2.3)	0.42
Time to surgery (months)			
From GLP-1 agonist initiation	6.3 (4.0)	–	**0.03**
From initial surgery clinic visit	7.6 (4.4)	14.7 (17.6)	0.06

Bold values indicate statistical significance

*GLP-1* glucagon-like peptide-1,* BMI* body mass index,* %TWL* percentage total weight loss,* SD* standard deviation

**Fig. 1:**
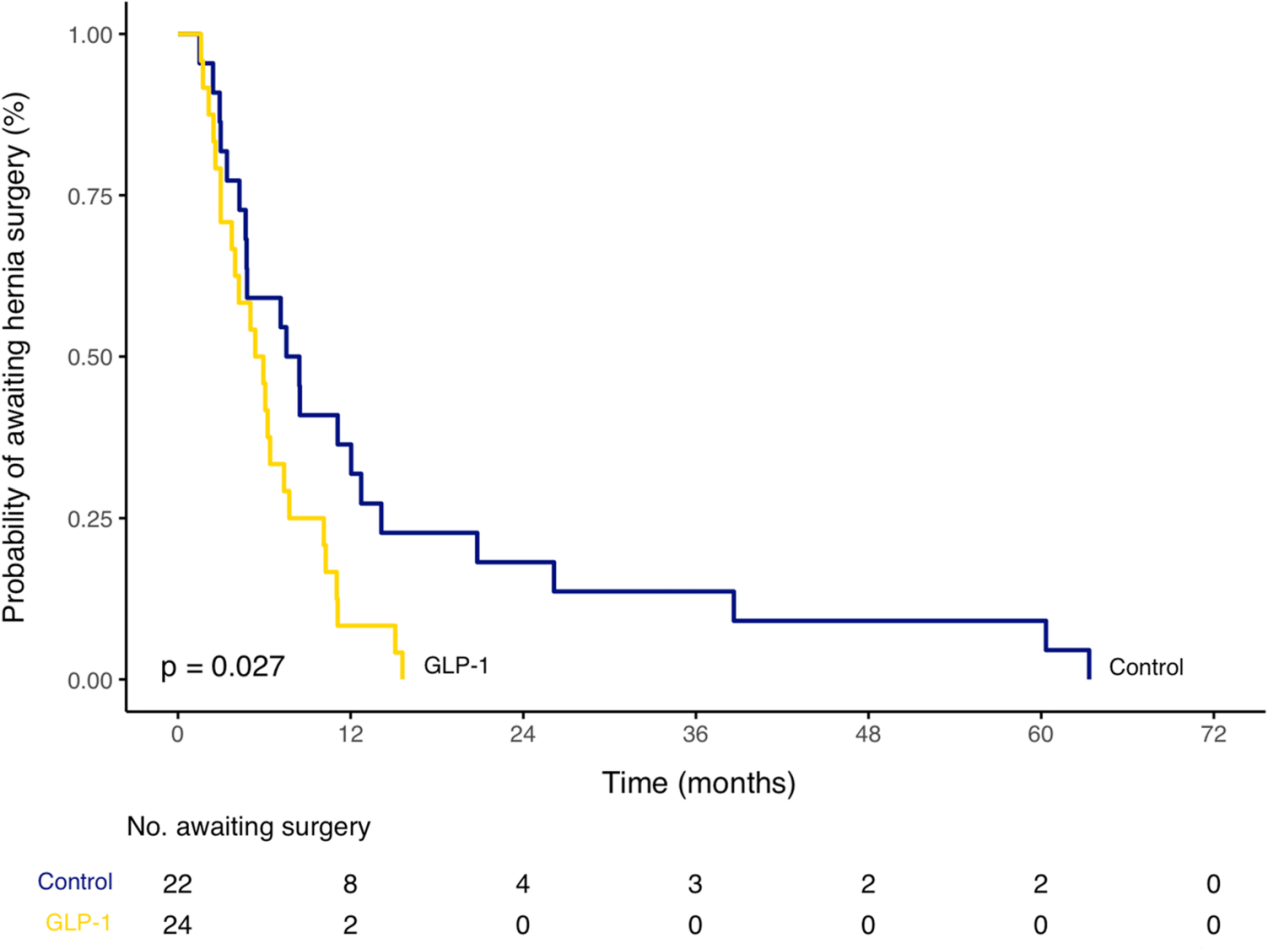
Kaplan–Meier plot of time to hernia surgery

The mean time from GLP-1 agonist initiation to surgery for the GLP-1 group was significantly shorter than the mean time from initial clinic visit to surgery for the control group (6.3 ± 4.0 months vs 14.7 ± 17.6 months, *p* = 0.03). A Kaplan–Meier time-to-event log-rank test revealed a significant difference in awaiting surgery risk favoring the GLP-1 group (Fig. 1). There was no statistically significant difference in the mean time from initial clinic visit to surgery between groups (7.6 ± 4.4 months vs 14.7 ± 17.6 months, *p* = 0.06) due to patients beginning GLP-1 therapy 1.3 months after initial clinic visit on average.

### Postoperative outcomes

The control group had a 27.3% 30-day morbidity rate compared to 8.3% for the GLP-1 group, although this difference was not statistically significant (*p* = 0.13). In the control group, there was 1 (4.5%) hernia recurrence and 1 (4.5%) 30-day reoperation for tissue flap necrosis. The GLP-1 group also had 1 (4.2%) 30-day reoperation for tissue flap necrosis and no hernia recurrences, although the control group had a significantly longer mean follow-up time (GLP-1: 4.2 ± 5.3 months vs control: 30.9 ± 28.3 months, *p* < 0.001). There were no 30-day mortalities. A summary of these results is detailed in Table 3. All reported postoperative outcomes are 30-day outcomes except for hernia recurrence.

**Table 3:** Postoperative outcomes

Outcome, *n* (%)	GLP-1 (*N* = 24)	Control (*N* = 22)	*p*-value
30-day morbidity	2 (8.3)	6 (27.3)	0.13
Superficial SSI	1 (4.2)	0	
Seroma	0	1 (4.5)	
Postoperative ileus	0	2 (9.1)	
Deep vein thrombosis	0	1 (4.5)	
Urinary tract infection	0	1 (4.5)	
Tissue flap necrosis	1 (4.2)	1 (4.5)	
30-day mortality	0	0	–
30-day reoperation	1 (4.2)	1 (4.5)	0.99
Hernia recurrence	0	1 (4.5)	0.99
Follow-up time (months), mean (SD)	4.2 (5.3)	30.9 (28.3)	** < 0.001**

Bold values indicate statistical significance

*GLP-1* glucagon-like peptide-1,* SSI* surgical site infection

## Discussion

Obesity is recognized as a significant risk factor for abdominal wall hernia recurrence and complications after hernia repair. It is therefore important to counsel patients on the importance of achieving weight loss goals before surgery to reduce complications and optimize the likelihood of a durable repair. However, weight loss with lifestyle modifications alone is often difficult, and many patients are lost to follow-up after initially being denied surgery due to high BMI [20]. GLP-1 agonists have rapidly gained popularity in the general population since their recent FDA approval for weight loss. In this study, we demonstrate that GLP-1 agonists enable effective weight loss in a cohort of 24 obese hernia patients and reduce time to surgery compared with lifestyle modifications alone without increasing postoperative complications. To our knowledge, this is the first study analyzing the impact of GLP-1 agonists on this patient population.

The results of this study highlight the potential of GLP-1 agonists to transform the prehabilitation of obese hernia patients. Compared to the control group, GLP-1 patients achieved similar preoperative weight loss in terms of %TWL and BMI reduction, with a time to surgery that was several months shorter on average. Although we did not demonstrate a statistically significant difference in average time to surgery from initial clinic visit between groups, a difference of 7.1 months likely represents a clinically significant difference for patients awaiting elective hernia repair. The ability of GLP-1 agonists to accelerate preoperative weight loss without increasing complications could possibly increase patient retention in hernia programs, enabling more patients to reach surgery than otherwise possible with lifestyle modifications alone. Increasing access to safe and durable hernia surgery may also decrease healthcare costs in the long-term by preventing hospital admissions for hernia-related complications and reducing the incidence of emergent hernia surgery. It’s possible that reducing the morbidity and prolonged hospital stays associated with emergent hernia surgery, in addition to the subsequent risk of hernia recurrence and need for reoperation, may provide a net cost reduction. As the use of GLP-1 agonists becomes more widespread in surgical practice, further studies are warranted to evaluate the cost-effectiveness of these medications and their impact on the development and performance of hernia programs more completely.

Despite being effective for many individuals, several factors exist that may limit the utility and availability of GLP-1 agonists for hernia patients. It has been recognized for several years that GLP-1 agonists have a variable and often unpredictable weight loss effect that is not fully understood. Proposed mechanisms for this variability include pharmacokinetic factors, polymorphisms in the GLP-1 receptor, and variability of central nervous system food-related responses to GLP-1 agonist action [21]. Responses also may vary due to age, gender, ethnicity, BMI, and comorbidities [22–25]. Early weight loss in treatment however has been identified as a key predictor of treatment success [26]. Further studies are needed to identify factors that may predict a GLP-1 weight loss response to potentially tailor hernia prehabilitation for individual patients.

In addition, insurance companies often restrict access to GLP-1 agonists due to cost. For example, the list price for Ozempic® at the time of drafting this manuscript is $935.77 for a 1-month supply [27]. The high cost of these medications without insurance coverage leaves many patients with few options and may motivate some to purchase the drugs in alternate markets. Recent shortages of GLP-1 agonists due to increased demand have also limited patient access. According to a recent insurance claims report, quarterly prescriptions of GLP-1 agonist surged by 300% between early 2020 and the end of 2022 [28]. US health care providers wrote more than 9 million prescriptions for GLP-1 agonists in the last 3 months of 2022 alone. The availability of GLP-1 agonists for on- and off-label use going forward will likely depend on manufacturer supply, drug cost, and insurance plan criteria for coverage. However, identification of a measurable benefit of GLP-1 agonists in terms of reducing hernia-related complications and increasing access to surgery may support the broad use of these drugs in hernia programs and potentially increase drug access. The development of guidelines for GLP-1 agonist use in hernia prehabilitation specifically would be beneficial as well.

The side effects of GLP-1 agonists are also an important factor. GLP-1 agonists are often associated with nausea, vomiting, diarrhea, and injection site reactions [29]. More severe, though less common, side effects of severe hypoglycemia and pancreatitis have reported rates of less than 1% in large clinical trials [30]. Although we did not formally assess for the incidence of side effects in this study, we did not detect any GLP-1 agonist discontinuation initiated by patients due to side effects. Nevertheless, close monitoring of side effects and appropriate dosage adjustment will be critical as GLP-1 agonist usage increases in perioperative weight management. Additionally, the risk of retained gastric contents and subsequent aspiration should be accounted for in all patients during the immediate perioperative period [31].

We continue to enroll patients in our hernia prehabilitation program and prescribe GLP-1 agonists due to these encouraging results and positive patient feedback. To date, we have enrolled 64 patients, including the 24 in this analysis that have undergone surgery. 28 patients (43.8%) remain in active weight loss follow-up with GLP-1 treatment. A total of 11 patients (17.2%) have been lost to follow-up, defined as no follow-up visit in at least 1 year. We plan to continue monitoring our long-term outcomes in terms of postoperative weight gain and hernia recurrence in subsequent studies.

There are several limitations to our study, namely its retrospective, single-center design with a limited sample size and follow-up time. Given that access to GLP-1 agonists is highly dependent on insurance coverage and financial resources, there may be socioeconomic factors biasing the composition of each patient cohort and therefore the ability of patients in each cohort to reliably follow-up. The timing of surgery for each patient also depends on surgeon availability, which can be influenced by multiple factors, such as the time of year and patient loads. It is also not clear how long patients should continue GLP-1 therapy postoperatively, and we plan to investigate this question in future studies. We recognize that including various hernia types introduces complexity into our analysis, however, we believe our heterogeneous patient population supports the utility of GLP-1 therapy in a variety of hernias and enhances the generalizability of our findings. Further large-scale clinical trials are warranted to evaluate the safety and efficacy of GLP-1 agonists in the perioperative weight management of surgical patients more broadly and to assess the impact of these medications on long-term hernia-related outcomes.

## Conclusion

GLP-1 agonists accelerate preoperative weight loss for obese hernia patients without negatively impacting postoperative outcomes. Further studies are warranted to evaluate the efficacy and cost-effectiveness of GLP-1 agonists in the perioperative weight management of surgical patients. 
